# Biotechnology of *Rhodococcus* for the production of valuable compounds

**DOI:** 10.1007/s00253-020-10861-z

**Published:** 2020-09-12

**Authors:** Martina Cappelletti, Alessandro Presentato, Elena Piacenza, Andrea Firrincieli, Raymond J. Turner, Davide Zannoni

**Affiliations:** 1 Department of Pharmacy and Biotechnology (FaBiT), University of Bologna, Bologna, Italy; 2 Department of Biological, Chemical and Pharmaceutical Sciences and Technologies (STEBICEF), University of Palermo, Palermo, Italy; 3 National Interuniversity Consortium of Materials Science and Technology (INSTM), Florence, Italy; 4 Department of Biological Sciences, Calgary University, Calgary, AB Canada

**Keywords:** *Rhodococcus*, Antimicrobials, Bioflocculants, Biosynthesis, Bioconversion, Biosurfactants, Carotenoids, Lipids, Metal-based nanostructures, Siderophores

## Abstract

**Abstract:**

Bacteria belonging to *Rhodococcus* genus represent ideal candidates for microbial biotechnology applications because of their metabolic versatility, ability to degrade a wide range of organic compounds, and resistance to various stress conditions, such as metal toxicity, desiccation, and high concentration of organic solvents. *Rhodococcus* spp. strains have also peculiar biosynthetic activities that contribute to their strong persistence in harsh and contaminated environments and provide them a competitive advantage over other microorganisms. This review is focused on the metabolic features of *Rhodococcus* genus and their potential use in biotechnology strategies for the production of compounds with environmental, industrial, and medical relevance such as biosurfactants, bioflocculants, carotenoids, triacylglycerols, polyhydroxyalkanoate, siderophores, antimicrobials, and metal-based nanostructures. These biosynthetic capacities can also be exploited to obtain high value-added products from low-cost substrates (industrial wastes and contaminants), offering the possibility to efficiently recover valuable resources and providing possible waste disposal solutions. *Rhodococcus* spp. strains have also recently been pointed out as a source of novel bioactive molecules highlighting the need to extend the knowledge on biosynthetic capacities of members of this genus and their potential utilization in the framework of bioeconomy.

**Key points:**

*• Rhodococcus possesses promising biosynthetic and bioconversion capacities.*

*• Rhodococcus bioconversion capacities can provide waste disposal solutions.*

*• Rhodococcus bioproducts have environmental, industrial, and medical relevance.*

**Figure:**
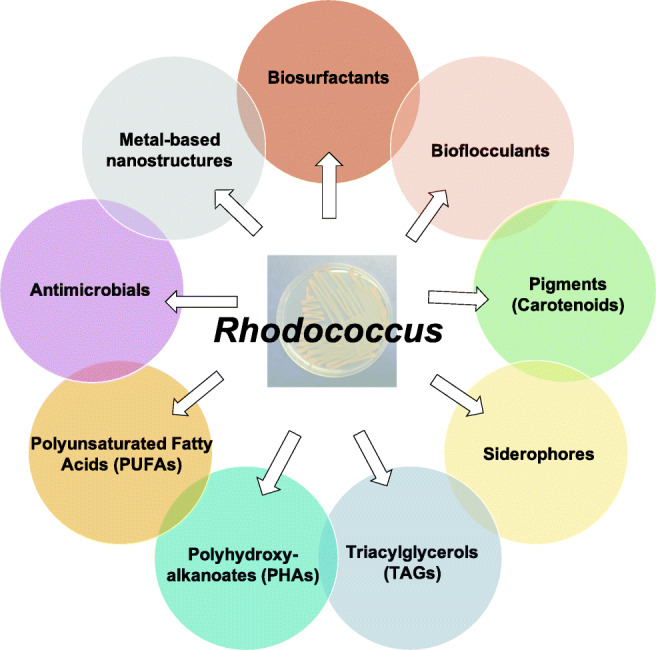

## Introduction

*Rhodococcus* genus belongs to the *Actinobacteria* phylum and comprises genetically and physiologically diverse bacteria, which are distributed in various water, soil, and marine habitats, also including harsh ecological niches such as arctic, desert, and heavily contaminated sites (Cappelletti et al. 2019a, 2019b). The wide distribution of *Rhodococcus* spp. strains is due to their extraordinary metabolic versatility, which is comparable to that described only in a few other bacterial genera, and to their unique environmental persistence and robustness (LeBlanc et al. 2008; Cappelletti et al. 2016).

Bacterial strains belonging to *Rhodococcus* genus have been largely studied for their application in bioremediation, biotransformation, and biocatalysis due to their capacity to biodegrade a wide range of organic compounds, including toxic and recalcitrant molecules such as chlorinated aliphatic and aromatic hydrocarbons, N- and S-heterocyclic compounds, and synthetic polymers (e.g., polyethylene) (Martínková et al. 2009; Cappelletti et al. 2015, 2017, 2018; Ciavarelli et al. 2012; Krivoruchko et al. 2019). During the last decade, it has become obvious that various species of this genus have also interesting capabilities regarding the biosynthesis of lipids and other valuable compounds. These biosynthetic capacities are often associated to the ability of *Rhodococcus* to resist to different environmental stresses (LeBlanc et al. 2008; Orro et al. 2015; Cappelletti et al. 2016) and allow these bacteria to cope with the presence of hydrophobic substrates in the growth medium, limited nitrogen sources, oxidative stressors, among others. The most extensively reported compounds that are biosynthesized by *Rhodococcus* spp. strains are glycolipid biosurfactants, carotenoids, triacylglycerols, and polyhydroxyalkanoates (PHAs) (Krivoruchko et al. 2019). Recently, novel siderophores and new antibiotics have been reported to be produced by *Rhodococcus* spp. strains as possible competition strategies. Further, several *Rhodococcus* spp. strains have recently been described to be able to produce metal-based nanostructures as the result of metal and metalloid [metal(loid)] bioconversion into their elemental state (Presentato et al. 2016, 2018a, 2018b; Firrincieli et al. 2019).

Compared to the many papers summarizing the biotechnological potential of *Rhodococcus* cells in association with their biodegradative enzymes for bioremediation and biocatalysis (Martínková et al. 2009; Kim et al. 2018; Krivoruchko et al. 2019; Jiao et al. 2020), this review gives an overview on the relevant biosynthetic capacities of bacteria belonging to this genus. These anabolic functions are at the basis of *Rhodococcus*-mediated bioproduction of commercially valuable compounds and bioactive molecules, as well as bioconversions of organic and inorganic contaminants/wastes into high value-added products.

## Biosurfactants

Biosurfactants are a heterogeneous group of amphiphilic surface-active compounds that are produced by various microorganisms including bacteria, yeast, and fungi and find wide applications in several industries as demulsifiers; wetting, foaming, and spreading agents; functional food ingredients; and soaps/detergents (Santos et al. 2016). Biological compounds with surfactant activities can generally be classified into low-molecular-weight (glycolipids, lipopeptides, and phospholipids) and high-molecular-weight (polysaccharides, proteins, lipoproteins, or polymeric compounds) substances, the first being considered actual biosurfactant and the second generally described as bioemulsifiers (Franzetti et al. 2010). They possess several advantages as compared to the chemical surfactants including biodegradability, lower critical micelle concentrations (CMC), reduced toxicity, higher stability, and the possibility to be produced by renewable raw material (Banat et al. 2010).

Several *Rhodococcus* spp. strains produce biosurfactants in response to the presence of water-insoluble substrates (e.g., hydrocarbons) (Whyte et al. 1999; Cappelletti et al. 2019a), and in some cases also on water-soluble substances (e.g., ethanol and glucose) (Table 1) (Pirog et al. 2004; Ciapina et al. 2006). The formation of biosurfactants is believed to improve the utilization (bioavailability) of these water-insoluble compounds as growth substrates by facilitating their entry into cells (Lang and Philp 1998; Yakimov et al. 1999; Philp et al. 2002; Peng et al. 2007; Cappelletti et al. 2019b).

**Table 1:** Biosynthetic activities of the most representative *Rhodococcus* spp. strains and growth substrate or waste residue used for the production of each valuable compound category

Product category	Strain/s	Substrate and/or growth conditions^a^	Features of the product and/or biosynthetic process	Reference
Biosurfactants	*R. erythropolis* DSM 43215	Cells growing on C_14_–C_15_ *n*-alkanes or kerosene	Production of extracellular trehalose-monomycolates and trehalose-dimycolates	Kim et al. 1990
	*Rhodococcus* H13-A	Cells growing on *n*-alkanes or fatty alcohols	Production of glycolipid which is able to solubilize polyaromatic hydrocarbons	Page et al. 1999
	*R. opacus* 1CP	Cells growing on C_10_, C_12_, C_14_, C_16_ *n*-alkanes	Production of trehalose dinocardiomycolates with double bonds	Niescher et al. 2006
	*R. erythropolis* ATCC 4277	Cells growing on glycerol or *n*-hexadecane	Production of extracellular biosurfactant on glycerol, and cell-bound biosurfactant on *n*-hexadecane	Ciapina et al. 2006
	*R. erythropolis* strain EK-1	Cells growing on hydrocarbons (C_16_ *n*-alkane and paraffin), ethanol or glucose	Production of cell-bound non-ionic trehalolipids on hydrocarbons, production of bioemulsifier on soluble substrates	Pirog et al. 2008
	*R. erythropolis* SD-74	Cells growing on *n*-hexadecane	Production of succynoil trehalolipids	Tokumoto et al. 2009
	*R. erythropolis* 3C-9	Cells growing on *n*-hexadecane	Production of extracellular trehalose lipids and free fatty acids	Peng et al. 2007
	*R. erythropolis* 16 LM.USTHB	Cells growing on sunflower frying oil	Production of extracellular glycolipids	Sadouk et al. 2008
	*Rhodococcus* sp. BS32	Cells growing on rapeseed oil	Production of extracellular biosurfactants	Ruggeri et al. 2009
	*R. erythropolis* sp. P6-4P	Cells growing on fish waste compost	Production of biosurfactant that is mainly composed of fatty acids	Kazemi et al. 2009
Bioflocculants	*R. erythropolis* S-1	Cells growing on sorbitol, mannitol, ethanol, glucose, or fructose	Production of the peptidic bioflocculant named NOC-1, it is one of the best performing bioflocculant described up to date	Kurane et al. 1994
	*R. erythropolis* ATCC 10543	Cells growing on pre-treated sludge and livestock wastewater	Production of a polysaccharidic bioflocculant	Peng et al. 2014
	*R. erythropolis*	Cells growing on potato starch wastewater	Production of a polysaccharidic bioflocculant	Guo et al. 2018
Carotenoids	*R.luteus*, *R. coprhilus*, *R. lentifragmentus*, *R. maris*	Cells growing on Sauton agar medium	Production of β-carotene	Ochiyama et al. 1989
	*R. equi*, *R. rubroperctinctus*, *R. aichiensis*, *R. sputi*, *R. chubuensis*, *R. obuensis*, *R. bornchialis*, *R. roseus*, *R. rhodochrous*, *R. rhodnii*, *R. terrae*	Cells growing on defined medium l-asparagine and glycerol as main nitrogen and carbon sources, respectively	Production of γ-carotene-like compound	Ochiyama et al. 1989
	*R. erythropolis* IBBPo1	Cells growing on *n*-alkane	Production of lycopene at higher level	Stancu et al. 2015
	*R. rhodochrous* RNMS1	ND	Production of γ-carotene derivatives	Takaichi et al. 1990
	*R. erythropolis* AN12	Cells growing on rich medium (i.e., nutrient broth-based medium)	Production of γ-carotene derivatives	Tao et al. 2004
	*Rhodococcus* sp. CIP	Cells growing on rich medium	Production of OH-chlorobactene glucoside hexadecanoate and related rare carotenoids	Osawa et al. 2011
	*R. opacus* PD630	Glycerol and ammonium acetate as main carbon and nitrogen sources, respectively	Production of uncharacterized carotenoids	Thanapimmetha et al. 2017
	*R. pyridinivorans* NT2	Cells growing on 2,4-dinitrotoluene	Production of a carotenoid mixture composed by 4-keto- γ-carotene and γ -carotene, lycopene, and β-carotene	Kundu et al. 2015
Triacylglycerols (TAGs)	*R. opacus* PD630	Cells growing on phenyldecane	Accumulation of TAGs containing phenyldecanoic acid residues	Alvarez et al. 2002
		Cells growing on *n*-alkane	Accumulation of fatty acids with the same carbon skeleton of the alkane used for growth	Alvarez 2003
		Cells growing on citrate, succinate, propionate, valerate, saturated, and mono-unsaturated fatty acids with C_15_ and C_17_ chain length	Accumulation of TAGs with increased fraction of odd-numbered fatty acids	Alvarez et al. 1997
		Cells growing on C_15_–C_18_ *n*-alkanes, phenylacetic acid, phenyldecane, gluconic acid, acetic acid, propionic acid, fructose, and olive oil	First work reporting the ability of PD630 strain to produce and accumulate TAGs using different carbon sources	Alvarez et al. 1996
		Cells growing on sugar beet molasses, and sucrose	Production of large amounts of TAGs was demonstrated using a high-cell-density cultivation in a 500-l pilot-plant scale	Voss and Steinbüchel 2001
	*R. opacus* MITXM-61 (i.e., the spontaneous mutant of *R. opacus* PD630 carrying exogenous genes of *xylA* and *xylB* from *Streptomyces padanus*)	Cells growing on xylose/glucose, or corn stover hydrolysates	Robust growth and TAG biosynthesis on high concentrations of xylose and simultaneous utilization of xylose and glucose in the corn stover hydrolysate	Kurosawa et al. 2013, 2014
	*R. opacus* PD630 pEC-K18*mob2*::*bglABCTF*	Cells growing on glucose and cellobiose	Acquisition of cellobiose utilization for growth and TAG accumulation	Hetzler and Steinbüchel 2013
	*R. opacus* MITGM-173 (i.e., adaptively evolved *R. opacus* PD630)	Cells growing on glycerol/glucose/xylose	Higher glycerol utilization as compared to the parental strain	Kurosawa et al. 2015b
	*R. erythropolis* strains DSMZ 43060, 17 and DM1–21; *R. fascians* strains F7, S1.17b, 123 and D188–5; *R. opacus* strains PD630 and MR22; *R. jostii* strains RHA1 and 602; *R. equi* ATCC 6939; *R. opacus* PD630 pTip-QC2 /*glpK1D1*_*F7*_	Cells growing on glycerol	*Rhodococcus* strains differed in terms of number of days required for TAGs production; *R. equi* produced lower amounts of TAGs as compared to the other species; *R. opacus* PD630 improved TAG production when expressing *glpK1D1* from *R. fascians*	Herrero et al. 2016
	*Rhodococcus* sp. 602	Cells growing on gluconate, benzoate, *n*-hexadecane, naphthalene, or naphthyl-1-dodecanoate	TAGs with fatty acids of different chain length were accumulated depending on the growth substrate, also under resting cell condition	Silva et al. 2010
	*R. jostii* LGK (*R. jostii* RHA1 bearing plasmid pTAC-*lgk*)	Cells growing on glucose, levoglucosan	RHA1 expressing *lgk* gene from *Lipomyces starkeyi* YZ-215 acquired the capacity to utilize levoglucosan for growth and TAG accumulation	Xiong et al. 2016a
	*R. jostii* RHA3 (*R. jostii* RHA1 bearing both *araBAD* and *araFGH*)	Cells growing on arabinose	RHA1 expressing *araBAD* and *araFGH* from *E. coli* acquired the capacity to utilize l-arabinose for growth and TAG accumulation	Xiong et al. 2016b
	*R. opacus* strains PD630 and MR22, *R. wratislaviensis* V, *R. jostii* RHA1, *R. erythropolis* DSMZ 43060, *R. fascians* F7, *R. equi* ATCC 6939, *R. jostii* RHA1 pJAM2/*ltp*1	Cells growing on olive mill wastes	*R. opacus*, *R. wratislaviensis*, and *R. jostii* were more efficient at producing cell biomass and lipids than *R. fascians*, *R. erythropolis*, and *R. equi.* The overexpression of *ltp1* (a gene encoding a fatty acid importer) in *R. jostii* RHA1 promoted an increase of 3.4-fold in lipids production	Herrero et al. 2018
	*Rhodococcus* sp. YHY01	Cells growing on oil palm biomass and barley straw lignin	Utilization of various aromatic compounds derived from lignocellulosic biomasses for growth and TAG accumulation	Bhatia et al. 2017, 2019
Polyhydroxyalkanoate (PHAs)	*R. ruber* NCIMB 40126	Cells growing on acetate, lactate, succinate, fructose, glucose, and molasses	Accumulation of PHA with 3 HV and 3HB monomers. The relative amount of each monomer changed depending on the substrate, but HV was generally the predominant	Haywood et al. (1991)
	*R. ruber* NCIMB 40126	Cells growing on 4-hydroxybutyrate and 1,4-butanediol	Accumulation of PHA mainly composed by 3 HV and 3HB but also incorporating 4HB	Haywood et al. (1991)
	*R. ruber* NCIMB 40126	Cells growing on 5-chlorovalerate	Accumulation of PHA mainly composed by 3 HV and 3HB but also incorporating 5HB	Haywood et al. (1991)
	*R. ruber* NCIMB 40126	Cells growing on hexanoate or 2-hexenoate	Production of PHA containing 3HB, 3 HV, and 3-hydroxyhexanoate (3HHx) monomer units	Haywood et al. (1991)
	*R. ruber* NCIMB 40126	Cells growing on valeric acid and 2-pentenoic acid	Accumulation of almost pure poly(3-hydroxyvalerate)	Haywood et al. (1991)
	*R. ruber* NCIMB 40126	Cells growing on glucose and valerate	Accumulation of the copolymer poly(3HB-co-3 HV) as main PHA	Alvarez et al. 1997b
	*R. rhodochrous* ATCC 19070	Cells growing on acetate, lactate, fructose, glucose, and sucrose	Accumulation of PHA with 3 HV and 3HB monomers. The relative amount of each monomer changed depending on the substrate, but HV was generally the predominant	Haywood et al. (1991)
	*R. erythropolis* DSMZ 43060*, R.fascians* D188–5*, R. opacus* MR22	Cells growing on glucose, gluconate, and valerate	Accumulation of small amounts of homopolymer PHB	Alvarez et al. 1997b
	*R. aetherivorans* IAR	Cells growing on toluene	Accumulation of the copolymer poly(3HB-co-3 HV) as main PHA	Hori et al. 2009
Polyunsaturated fatty acids (PUFA)	*R. aetherivorans* BCP1	Cells growing on cyclopentane carboxylic acid, cyclohexane carboxylic acid, and glucose	Production of linoleic acid increased on CPCA as compared to the other C sources	Presentato et al. 2018a
Antimicrobials	*R. jostii* K01-B0171	Cells growing on rich medium with mannitol, glucose, yeast extract, ammonium succinate as carbon and nitrogen sources	Production of lariantin A and B	Inokoshi et al. (2012)
	*R*. *erythropolis* JCM 6824	Cell growing on succinate, sucrose, and casamino acids	Production of aurachin RE	Kitigawa et al. (2018)
	*Rhodococcus* sp. Acta 2259	Cells growing in rich medium	Production of four aurachins	Nachtigall et al. 2010
	*Rhodococcus* sp. Mer-N1033	Cells growing in rich medium	Production of rhodopeptins	Chiba et al. 1999
	*R. fascians* 307CO	Co-culturing with *Streptomyces padanus*	Production of rhodostreptomycin A and B	Kurosawa et al. 2008
	*R. erythropolis* JCM 2895	Soft-agar assay with rich medium medium	Production of a bacteriocin-like molecule	Kitigawa et al. 2018
	*R. equi* and *R. erythropolis* strains, *Rhodococcus enclensis* NIO-1009	Genome-based analyses	Production of humimycin A and B	Chu et al. 2016
Siderophores	*R. erythropolis* IGTS8	Cells growing in defined salt media under iron limitation conditions	Production of heterobactin A and B	Carrano et al. 2001
	*R. erythropolis* PR4	Cells growing on glucose, under iron-depleted conditions	Production of heterobactin A, heterobactins S1 and S2	Bosello et al. 2013
	*R. jostii* RHA1	Cells growing on glucose under iron-depleted conditions	Production of rhodochelin	Bosello et al. 2011
	*R. rhodochrous* OFS	Cells growing on hexadecane in iron-deficient minimal medium	Production of rhodobactin	Dhungana et al. 2007
	*R. erythropolis* S43	Cells growing on glucose, under iron-depleted conditions	Production of siderophore biding trivalent arsenic, AsO_3_^3-^ [also referred to as As(III)]	Retamal-Morales et al. 2018b
Metal(loid) nanomaterials	*Rhodococcus* sp. strain	Resting cells and HAuCl_4_ as precursor	Production of intracellular crystalline AuNPs (ca. 12 nm)	Ahmad et al. 2003
	*Rhodococcus* sp. NCIM2891	Cells growing using sodium acetate or cell-free extracts, AgNO_3_ as precursor	Production of intracellular crystalline AgNPs (ca. 10 nm)	Otari et al. 2012
	*R. pyridinivorans* NT2	Cells growing in rich medium or cell-free extracts; ZnSO_4_·H_2_O as precursor	Production of extracellular spherical and hexagonal crystalline ZnO NPs (ca.100 nm)	Kundu et al. 2014
	*R. aetherivorans* BCP1	Cells growing in rich medium; TeO_3_^2−^ as precursor	Production of intracellular TeNRs (from 100 to 500 nm)	Presentato et al. 2016
		Resting cells; TeO_3_^2−^ as precursor	Production of intracellular crystalline TeNPs and TeNRs (from 200 to 700 nm)	Presentato et al. 2018b
		Cells growing in rich medium; SeO_3_^2−^ as precursor	Production of SeNPs and SeNRs (from 50 to 600 nm)	Presentato et al. 2018a
	*R. erythropolis* ATCC 4277	Cells growing in rich medium or in a stirred tank reactor; sulfate mineral coal tailings as precursor	Production of crystalline Fe_2_O_3_ NPs (ca. 50 or 100 nm)	Maas et al. 2019a, 2019b

^a^In C_*x*_, *x* indicates the number of carbons in the alkane chain

**Fig. 1:**
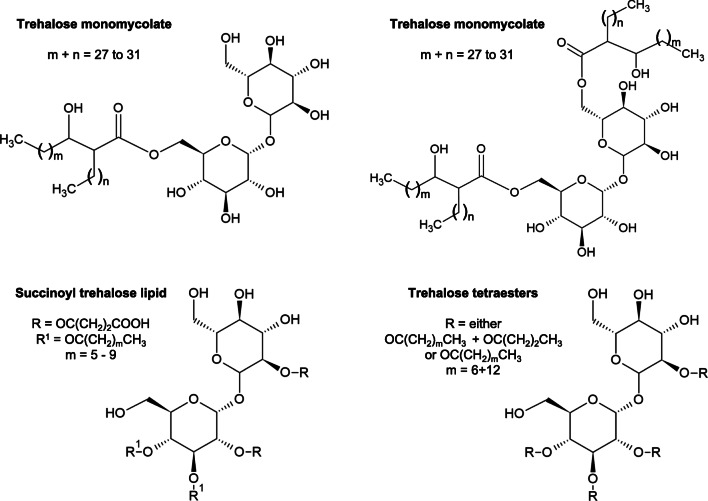
Chemical structures of the main biosurfactants produced by *Rhodococcus* spp. strains (modified from Franzetti et al. 2010; Kuyukina and Ivshina 2010)

Rhodococci typically produce trehalose-based glycolipid biosurfactants, which possess various hydrophobic moieties and chemical compositions (Kuyukina and Ivshina 2010) (Fig. 1). Trehalolipids (TP) are a class of glycolipid biosurfactants with interesting physicochemical and biological properties that have been studied for i) environmental applications as emulsifiers in bioremediation (e.g. oil-spill treatment), ii) microbial-enhanced oil recovery, and iii) cosmetic and food industries (Christofi and Ivshina 2002; Pacheco et al. 2010). Besides, these compounds can be used for medical application for their immune-stimulating, antitumor, and antiviral properties (Ortiz et al. 2008; Kuyukina et al. 2015).

TP are generally produced by rhodococci as either extracellular or cell wall-associated compounds during the growth on hydrocarbons (Lang and Philp 1998; Yakimov et al. 1999). Nitrogen limitation was also shown to favor the formation of anionic trehalose tetraesters using these growth substrates (Ristau and Wagner 1983; Kim et al. 1990). In those *Rhodococcus* spp. strains where the biosurfactants are excreted from the cell, the emulsification of the hydrocarbons with aqueous culture medium results in a very large surface area of contact between the cell and these compounds. Nevertheless, TP are more frequently retained by the *Rhodococcus* cell and localized on the outer cell surface (Rapp et al. 1979), increasing the hydrophobicity of the cell surface that facilitates the attachment and subsequent uptake of the hydrophobic compounds to be used as growth substrate (Kretschmer and Wagner 1983; Bredholt et al. 1998; Lang and Philp 1998; Cappelletti et al. 2019a). These biosurfactants, which bind to the cells, have more limited industrial applications because of problems with their recovery. The mechanism involved in the production and retention of biosurfactants by *Rhodococcus* cells appeared to be related to the type of substrate and the cell growth phase (Bredholt et al. 2002). Particularly, a marine *Rhodococcus* strain grown in the presence of sunflower oil, produced trehalose-based biosurfactants that were bound to cells during the first growth phase, while a strong increase of extracellular trehalolipid level was observed after the onset of stationary growth phase, reaching a maximum level of ~75% of the total trehalolipids present in the liquid medium (White et al. 2013). Further, the formation of anionic trehalose ester in *R. opacus* 1CP was associated with both biomass growth and *n*-alkane consumption, whereas the trehalose lipid production by *R. erythropolis* DSM43215 seemed to be uncoupled from growth and occurred in the stationary phase or under resting cells condition (Kim et al. 1990). An additional aspect regards the substrate used for the biosurfactant production, as *R. erythropolis* ATCC 4277 produced extracellular glycolipids upon growth on glycerol as sole carbon source, while partially cell-bound biosurfactants were obtained when *n*-hexadecane was added as only carbon and energy source (Ciapina et al. 2006). The presence of hydrocarbons (liquid paraffin and hexadecane) favored the generation of cell-bound trehalose-based glycolipids in *R. erythropolis* EK-1 cells. Conversely, strain EK-1 produced extracellular bioemulsifiers when the cells were grown on soluble substrates (ethanol and glucose) (Pirog et al. 2008; Franzetti et al. 2010). These considerations highlight the wide diversity in biosurfactant production mechanisms among different *Rhodococcus* strains and the possibility to drive the production of specific surfactant molecules by selecting the growth substrate. In this regard, supplying *R. erythropolis* SD-74 cultures with different *n*-alkanes as carbon source led to the biosynthesis of succinoyl trehalolipids featuring acyl groups of the same carbon chain length as the growth substrate (Tokumoto et al. 2009), suggesting the possibility to direct the synthesis of specific biosurfactants by providing alkanes with specific chemical structure (Lang and Philp 1998; Inaba et al. 2013; Cappelletti et al. 2019a). This property is incredibly useful for the commercial production, as most biosurfactant producers do not synthesize specifically defined derivatives. On the other hand, different *Rhodococcus* spp. strains produced diverse biosurfactants using the same hydrocarbon as a carbon source. For instance, *Rhodococcus* sp. SD-74 generated extracellular succinoyl trehalose lipids when cultivated on *n*-hexadecane (Tokumoto et al. 2009), while the same substrate in *R. erythropolis* 3C-9 favored the synthesis and release of two types of biosurfactants, i.e., trehalose lipids and free fatty acids—the latter being rarely reported in *Rhodococcus* spp., with the exception of *R. ruber* (Kuyukina et al. 2001; Peng et al. 2007). Other active compounds with surfactant activity produced by *Rhodococcus* were extracellular polysaccharides (EPS), which, in the case of *R. rhodochrous* S-2, enhanced its tolerance to the aromatic fraction (AF) of crude oil and facilitated the growth of indigenous bacteria resulting in the promotion of AF degradation in seawater-based medium (Iwabuchi et al. 2002).

Biosurfactants produced by some *Rhodococcus* species were reported to be more efficient in reducing the surface and interfacial tensions between aqueous and oil phases and to have lower critical micelle concentrations (CMCs) than many synthetic surfactants (Lang and Philp 1998). For instance, the biosurfactant produced by *Rhodococcus* sp. strain H13-A was up to 35-fold more effective than the synthetic Tween 80 counterpart in solubilizing polyaromatic hydrocarbons from a complex mixture into an aqueous solution (Page et al. 1999). Furthermore, in several cases, the biosurfactants from *Rhodococcus* were also found to have extremely low toxicity, sometimes lower as compared to that of biosurfactants isolated from other bacterial genera. Indeed, biosurfactants produced by and extracted from *R. ruber* AC 235 were 100–1000 times less toxic than synthetic commercial surfactants (Inipol EAP22, Corexit 9597, Finasol OSR-5) and 13 times less toxic than rhamnolipids from *Pseudomonas aeruginosa* (Kuyukina et al. 2001).

To reduce the cost of producing biosurfactant with rhodococci, specific approaches, such as the exploitation of low-cost substrates, were used. Raw material accounts for almost 30% of the overall cost of a microbial surfactant production and the investigation on potential usage of low-cost raw materials (e.g., industrial and/or municipal wastes) is necessary to develop economically sustainable processes (George and Jayachandran 2013). In this context, *R. erythropolis* 16 LM.USTHB showed the capacity to convert residual sunflower frying oil, a cheap renewable substrate, into extracellular glycolipids, which effectively lowered the surface tension of the crude broth (Sadouk et al. 2008). Ruggeri et al. (2009) isolated *Rhodococcus* sp. BS32, a strain able to produce extracellular biosurfactants growing on rapeseed oil. Fish waste compost was an effective source of nutrient-rich organic matter for the growth of *R. erythropolis* sp. P6-4P and the production of biosurfactants (Kazemi et al. 2009).

Despite promising features of the biosurfactants produced by rhodococci as compared to the well-understood biochemical and molecular bases of rhamnolipids synthesized by *Pseudomonas* strains (George and Jayachandran 2013), less information is available for the genetic and biochemistry of TL generation in *Rhodococcus*. It seems that the hydrophilic (the trehalose moiety) and hydrophobic (the mycolic acid moiety) portions of the trehalolipid molecules are synthesized independently and are subsequently esterified (Kretschmer and Wagner 1983; Kuyukina and Ivshina 2010). During biosynthesis, trehalose is first esterified with mycolic acids synthesized in the cytoplasm to form the trehalose monomycolate, which is believed to be the precursor of the di- or trimycolates produced at the plasma membrane (Lang and Philp 1998). Moreover, an enoyl-acyl carrier protein reductase, InhA, was indicated to be part of the fatty acid synthase-II system (FASII) involved in the process of the elongation of medium-sized fatty acids leading to long-chained hydrophobic moieties of trehalose mycolates in *Rhodococcus* spp. (Asselineau et al. 2002). The trehalose-6-phosphate synthase, OtsA, was reported to be the key enzyme involved in the synthesis of the sugar residue of the trehalose lipids biosynthesis (Tischler et al. 2013). The presence of an alternative trehalose biosynthesis pathway mediated by maltooligosyltrehalose synthase (TreY) and maltooligosyltrehalose trehalohydrolase (TreZ) enzymes was predicted in the biosurfactant producer *R. erythropolis* B7g (Retamal-Morales et al. 2018a). This pathway for trehalose biosynthesis uses oligo/polymalto-dextrins/glycogen as substrate (Tropis et al. 2005).

## Bioflocculants

Flocculants are used as additives that induce aggregation or agglomeration of colloidal and other particles to form large particles (flocs) which settle allowing the clarification of the system in sedimentation and clarification processes (Lee et al. 2014). In this context, flocculants have been extensively applied for removing turbidity, suspended and dissolved solids, colors and dyes, and chemical oxygen demand (COD) in tap- and wastewater treatment processes. Compared with traditional inorganic and organic synthetic flocculants such as polyacrylamides, bioflocculants are more readily biodegradable, as well as less toxic to humans and environments (Salehizadeh et al. 2018).

Bioflocculants produced by *Rhodococcus* spp. strains received a large amount of attention due to their strong flocculating activities (Peng et al. 2014; Jiang et al. 2019). They were reported to mainly consist of polypeptide and lipid assemblies, especially mycolate-containing glycolipids (Finnerty 1992), which should allow the bacteria to more easily access the growth substrate. Specifically, bioflocculants seemed to be produced by those *Rhodococcus* spp. strains that do not synthesize biosurfactants during the utilization of long-chain alkanes (Bouchez-Naïtali et al. 2001). One of the first studies on this topic indicated that the production of an extracellular flocculant by *R. erythropolis* S-1 cells was strongly dependent on the type of substrate added. Although glucose, fructose, sorbitol, and mannitol were all efficiently used for bioflocculant production by S-1 strain, ethanol resulted to be the best performing carbon source for this purpose (Kurane et al. 1994), whereas organic acids and phenol did not induce any bioflocculant generation. The bioflocculant produced by S-1 resulted to be usable on a wide range of suspended solids, such as acid and alkaline soils, as well as India ink (Kurane et al. 1994). The same *Rhodococcus* strain could also use *n*-pentadecane as a carbon source, yet in this culture medium, the flocculant was found to be located on the cell surface, influencing the cell appearance during S-1 growth. Indeed, cells forming fibrous flocs that floated at the surface were observed upon S-l cultivation in the medium containing *n*-pentadecane, whereas when glucose was used as the carbon source, the cells were dispersed (Takeda et al. 1991). Characterization analyses performed on the bioflocculant produced by S-1 revealed that its main component was a protein (Kurane et al. 1994; Takeda et al. 1991). This bioflocculant was named NOC-1 and it has been used in both environmental pollution control and industrial wastewater treatment. *R. erythropolis* S-1 is currently recognized as one of the best flocculating bacteria and was used as a reference strain in studies on microbial flocculant production (Jiang et al. 2019).

Several studies investigated the possibility to reduce the cost of bioflocculant development, by using economical culture media for bacterial growth (Table 1). For instance, using pre-treated sludge and livestock wastewater as growth substrate, *R. erythropolis* ATCC 10543 produced a polysaccharidic bioflocculant featuring a strong flocculating potential on the same wastewaters within a wide pH range (2–12) (Peng et al. 2014). A recent study reported on the use of potato starch wastewater by another *R. erythropolis* strain to produce, parallelly to its growth, a bioflocculant, which showed a polysaccharide nature. The effectiveness of the bioflocculant was harnessed by treating the potato starch wastewater at neutral pH and analyzing the residual organics (COD and BOD), ammonium, phosphorus, turbidity, and chroma. This bioflocculant could be successfully applied to the potato starch wastewater treatment in a sequencing batch reactor (SBR), indicating that the compound was active in a real application scenario (Guo et al. 2018).

## Carotenoids

Carotenoids are pigments ranging in color from yellow to orange and deep red that are soluble in lipids. Chemically, they are usually tetraterpenoids composed of a 40-carbon atom polyene skeleton, which is either acyclic or terminated by one or two cyclic end groups. In nature, carotenoids protect cell membranes against damage by light, oxidation, and free radicals (Thanapimmetha et al. 2017); further, several carotenoids have been proved to play an important role in the prevention of human diseases and maintaining good health, due to antioxidant and anticancer properties (Rao and Rao 2007). Moreover, in humans and animals, α-carotene, β-carotene, and β-cryptoxanthin are precursors of vitamin A; thus, carotenoids are widely used as food additives, as well as pharmaceuticals, poultry, and cosmetic products (Saini and Keum 2017). Nowadays, there is an increasing demand for “natural” carotenoids to replace the chemically synthesized ones due to the impelling scientific interest towards green technologies and the public awareness about the possible toxicity of synthetic food additives. The microbial production of carotenoids is considered as a promising alternative, as carotenoid-rich microbial biomass can be grown in bioreactors using inexpensive substrates and under controllable culture conditions to maximize production (Agarwal and Rao 2000; Thanapimmetha et al. 2017).

Many *Rhodococcus* spp. strains can produce different types of carotenoid pigments (Table 1) (Takaichi et al. 1990; Tao et al. 2004), which are located either intracellularly (e.g., in lipid droplets or in the vicinity of the plasma membrane) or around the hydrophobic *Rhodococcus* cell wall. As they are non-photosynthetic bacteria, the role of carotenoids was generally associated with cell protection from various oxidative damages, as demonstrated by testing cell response to H_2_O_2_ treatment and to methylene blue-sensitized photo-oxidation (Osawa et al. 2011; Bequer Urbano et al. 2014). Further, the increase of carotenoid accumulation in these bacteria or the modification of carotenoid profile was associated with the type of growth and the presence of organic solvents. Indeed, carotenoids were suggested to have an antioxidant role in *Rhodococcus* cells grown as biofilm, whose development could induce an oxidative stress in individual cells within the community due to an increase of reactive oxygen species (ROS) levels (Zheng et al. 2013; Kundu et al. 2015). *Rhodococcus erythropolis* IBBPo1 cells exposed for 1 or 24 h to 1% alkanes instead showed a modification of the carotenoid profile with an increased level of lycopene (the intermediate in the biosynthesis of γ-carotene and β-carotene) as compared to the control, likely as a response to these organic solvents (Stancu 2015). Lastly, carotenoid formation in a *Rhodococcus* strain isolated from *Azolla* symbiotic cavities was reported to be induced by cells’ exposition to the light, which led to a sevenfold higher accumulation of a yellowish-orange pigment than dark-incubated cells (Cohen et al. 2004).

**Fig. 2:**
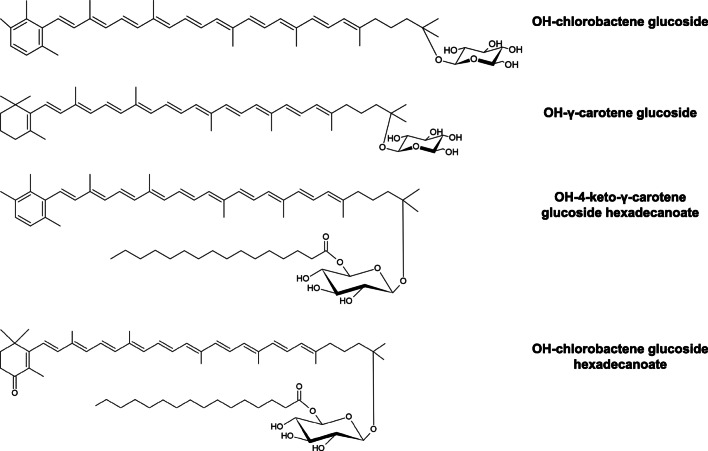
Chemical structures of the main carotenoids produced by *Rhodococcus* sp. 1CP

The first study on the ability of *Rhodococcus* strains to produce carotenoids was performed by Ichiyama et al. (1989), who cultivated 16 *Rhodococcus* strains, each belonging to a different species, using glycerol as the main carbon source, at pH 7 and at 37 or 28 °C (Ichiyama et al. 1988, 1989). Based on thin-layer chromatography, the strains produced 11 different carotenoid-type pigments, whose nature was only partly identified. Nevertheless, *Rhodococcus* species were divided into three groups depending on the type of pigment synthesized, i.e., β-carotene, a γ-carotene-like substance, or neither of these carotenes; some species also produced derivatives of β- and γ-carotene, such as myxoxanthophyll-like, zeaxanthin-like, and β-citraurin-like carotenoids. In this work, the biosynthesis of β-carotene was suggested to originate from a conversion of γ-carotene. The presence of the enzyme catalyzing the transformation of γ-carotene into β-carotene distinguished the species under analysis (Ichiyama et al. 1989). Among these, *R. maris* and *R. ruber* were subsequently reported as β-carotene (bicyclic carotenoid) producers without being further characterized (Ichiyama et al. 1989). On the other hand, *R. rhodochrous* RNMS1 and *R. erythropolis* AN12 were described to produce only monocyclic carotenoids such as γ-carotene derivatives (Takaichi et al. 1990; Tao et al. 2004). This aspect was related in the AN12 strain with the peculiar activity of the lycopene β-monocyclase encoded by the *crtLm* gene, which produced almost exclusively γ-carotene from lycopene. In relation to this, *Rhodococcus* is one of the few bacterial genera, which are able to produce only monocyclic carotenoids such as γ-carotene derivatives. More frequently, monocyclic carotenoids are part of the mixture formed during the synthesis of bicyclic carotenoids (e.g., β-carotene). The asymmetrically acting lycopene β-cyclase from AN12 strain is therefore of great biotechnological significance to obtain asymmetric carotenoids of commercial interest, which are generally very difficult to produce chemically (Tao et al. 2004). An additional work by Tao and Cheng (2004) further described the remaining of the carotenoid synthesis genes (*crt* genes) in AN12. Among these genes, *crtO* encodes a β-carotene ketolase, whose heterologous expression in an *Escherichia coli* strain accumulating β-carotene resulted in the production of canthaxanthin (β,β-carotene-4,4′-dione) (Tao and Cheng 2004), which is an orange-to-red keto-carotenoid exhibiting strong antioxidant activities and the ability to reduce and protect against UV light-induced tumor/types of cancers (Rao and Rao 2007). Additionally, members of genus *Rhodococcus* are among the few selected microbes producing carotenoids with conjugated keto functions (Ichiyama et al. 1989). In particular, keto-carotenoids are characterized by high polarity which facilitates their absorption and distribution once ingested and positively influences their antioxidant activity in membranes (Borroni et al. 2017). Recently, during a screening for antioxidative carotenoids of bacterial origin, a novel aromatic carotenoid (OH-chlorobactene glucoside hexadecanoate) and rare carotenoids (OH-chlorobactene glucoside, OH-carotene glucoside, and OH-4-keto-carotene glucoside hexadecanoate) were found to be produced by the orange-pigmented *Rhodococcus* sp. CIP isolated from a soil sample (Fig. 2). The produced carotenoids showed potent antioxidative activities, as indicated by analyzing the ^1^O_2_ quenching abilities of the compounds. The biosynthetic pathway for these promising carotenoids showed the γ-carotene as the main metabolic intermediate, which is further modified by the activities of sequential enzymes, such as a desaturase (CrtU), a hydroxylase (CrtC), and a glucosyltransferase (CruC), an acyltransferase (CruD), or a β-carotene ketolase (CtrO) (Osawa et al. 2011).

In the view of setting the bases for a possible industrial process, the carotenoid content of the biomass, the culture productivities, and biomass yields were evaluated in batch and fed-batch cultures of *R. opacus* PD630 using glycerol and ammonium acetate as cheap carbon and nitrogen sources, respectively. These experiments indicated that carotenoid production was dependent on the biomass growth, while no effect was observed upon modifications of the growth medium pH (Thanapimmetha et al. 2017). Under similar growth conditions, adding glucose as a co-substrate in a glycerol-based culture medium and controlling the nitrogen supply improved the production of biomass, lipid, and carotenoids. Particularly, a repeated fed-batch operation with controlled feeding of carbon- and nitrogen-substrates increased the productivity of carotenoids in PD630 up to 3.5-fold, and a 5-fold increase in the final lipid concentration (Suwaleerat et al. 2017). In addition to the use of glycerol as cheap substrate, organic contaminants are promising carbon sources for the sustainable production of carotenoids using *Rhodococcus* spp. strains. In this respect, a few studies have reported the capacity of *Rhodococcus* spp. strains to accumulate carotenoids growing on hydrocarbon and nitroaromatic compounds (Table 1) (Stancu 2015; Kundu et al. 2015).

## Intracellular accumulation of storage compounds

*Rhodococcus* spp. strains are able to synthesize and accumulate storage lipids in the form of polyhydroxyalkanoates (PHAs) (Anderson and Dawes 1990), triacylglycerols (TAGs) (Alvarez et al. 1996), or wax esters (WEs) (Lanfranconi and Alvarez 2017; Alvarez and Steinbüchel 2019) as an adaptation response to specific growth conditions and nutrients.

**Fig. 3:**
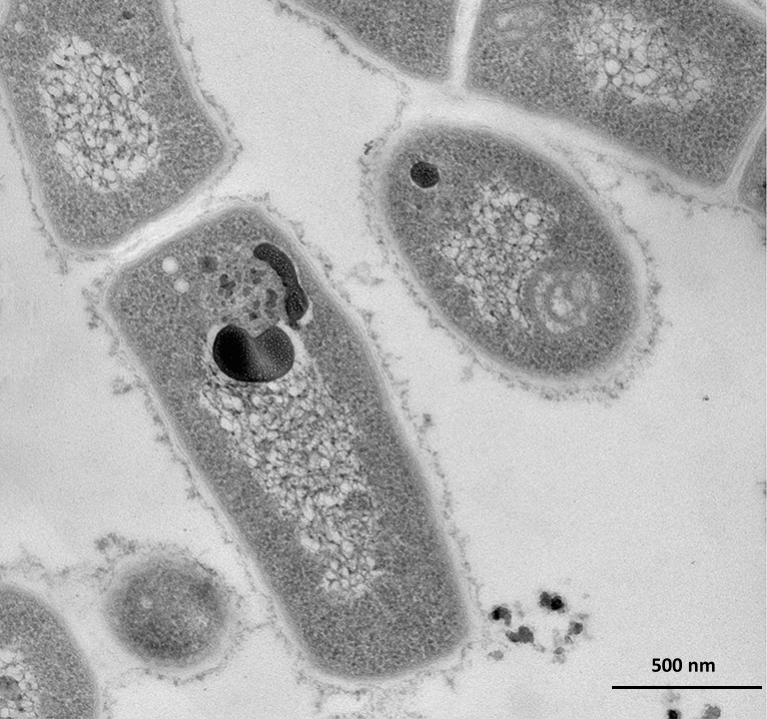
Transmission electron microscopy (TEM) image of *Rhodococcus aetherivorans* BCP1 cells grown on naphthenic acids. The electron-dense intracellular body represents a possible polyP granule (from Presentato et al. 2018a)

In addition to the accumulation of lipid-based molecule, *Rhodococcus* spp. can also produce inclusions enriched in polyphosphate (polyP) and/or carbohydrates (i.e., glycogen and trehalose) (Hernández et al. 2008). In this respect, the microbial polyP production has been described as a promising approach to remove or recover phosphate from wastewaters (Wang et al. 2018). PolyP granules are formed as a response to unfavorable environmental conditions, such as osmotic and oxidative stresses, low environmental phosphate load, desiccation, and heat shock. Indeed, oligotrophic growth conditions determined the accumulation in *R. erythropolis* N9T-4 of polyP granules, which were named oligobodies due to their analogies with acidocalcisomes of eukaryotic cells (Yoshida et al. 2017; Presentato et al. 2018a). Similar polyP granules were intracellularly generated by *R. aetherivorans* BCP1 cells upon utilization of cyclohexane carboxylic acid (CHCA, a naphthenic acid molecule) as the sole carbon and energy source (Fig. 3). Recently, the intracellular accumulation of polyP was proposed as an energy sink to replace ATP, which may have a regulatory effect towards specific enzymatic activities (Hernández et al. 2008). On the other hand, the accumulation of glycogen and trehalose in *Rhodococcus* was considered a carbon and energy storage strategy occurring in response to either N-limiting conditions of growth or hyperosmotic stress (Hernández et al. 2008).

### Triacylglycerols

The ability to accumulate triacylglycerols (TAGs, glycerol esters with long-chain length fatty acids) and wax esters (WE, esters of primary long-chain fatty acids and primary long-chain fatty alcohols) has been reported for only some bacterial genera including *Streptomyces*, *Rhodococcus*, *Mycobacterium*, and *Nocardia* (Alvarez et al. 2019). Bacterial TAGs were proposed to function as reserve compounds for carbon and energy, although other roles have been also discussed in relation to the regulation of cellular membrane fluidity and with the sink for reducing equivalents (Alvarez and Steinbuchel 2002). These lipids have relevant applications in the production of food additives, cosmetics, lubricants, oleochemicals, candles, and biofuels (Alvarez and Steinbüchel 2002). Alternative sources for TAG production are derived from the agriculture, although the advantages of using microorganisms over agricultural sources are numerous and mainly relating to the high variability of the fatty acid composition produced, as well as the better accessibility of microorganisms to genetic and metabolic engineering.

**Fig. 4:**
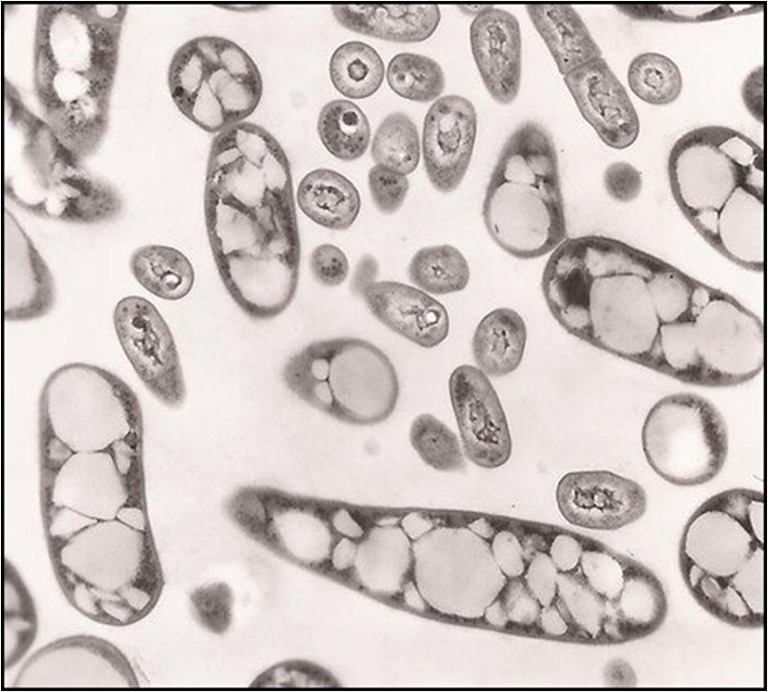
Transmission electron microscopy (TEM) image of *Rhodococcus opacus* PD630 cells containing TAG granules (from Alvarez et al. 2013)

Among the TAG accumulating genera, *Rhodococcus* is one of the most promising since bacterial strains of this genus can accumulate significant amounts of lipids (above 20% of the cell dry weight), being for this reason referred to as “oleaginous” (Table 1). In particular, *R. opacus* and *R. jostii* strains are considered oleaginous models as they produce large amounts of TAGs (up to 87% of the cellular dry weight in *R. opacus* PD630) (Fig. 4) (Alvarez et al. 1996; Alvarez and Steinbüchel 2002; Alvarez 2016), which are visible inside the cells as insoluble inclusions surrounded by a thin membrane (Alvarez et al. 1996; Alvarez 2003). They also showed the ability to accumulate unusual acyl moieties in TAGs, such as phenyldecanoic acid (Alvarez et al. 2002).

As compared to *R. opacus* and *R. jostii*, *R. fascians*, *R. erythropolis*, and *R. equi* generally produced lower amounts of TAGs during cultivation on sugars, organic acids, or hydrocarbons (Table 1) (Alvarez et al. 1997; Alvarez 2003; Herrero et al. 2018). The different biotransformation performance is due to genetic and physiological differences among *Rhodococcus* spp. strains. The high lipid production performance of *R. opacus*, *R. jostii*, and *R. wratilaviensis* was attributed to their large genomes and high number of genes coding for transporters and enzymes involved in the lipid metabolism (Cappelletti et al. 2019a). On the other hand, *R. fascians* and *R. erythropolis* were the most efficient species in TAG accumulation from glycerol. Despite the smaller genome, these *Rhodococcus* spp. possessed the genes *glpFK1D1*, which are involved in glycerol degradation and are absent in *R. opacus* and *R. jostii* (Herrero et al. 2016).

In addition to the species/strain itself, other factors influence the amount, composition, and structure of lipids in *Rhodococcus* such as the carbon source used, the time of cultivation, and the amount of carbon and nitrogen present in the culture medium (Alvarez et al. 1997; Wältermann et al. 2005). Different *Rhodococcus* spp. strains have shown the ability to produce and accumulate TAG using different substrates including defined carbons sources like sugars and organic acids (Alvarez et al. 1996, 1997, 2000; Silva et al. 2010), single organic contaminants (e.g., aliphatic and aromatic hydrocarbons) (Alvarez et al. 1997; Silva et al. 2010) but also complex carbon sources present in agro-industrial wastes, such as sugar beet molasses, orange, olive mill wastes, whey, oil palm biomass and corn stover (Voss and Steinbuchel 2001; Kurosawa et al. 2014; Bhatia et al. 2017; Herrero et al. 2018).

The growth substrate supplied to the culture medium influenced the amount and chemical nature of the accumulated TAGs. For instance, in *R. opacus* PD630 culture, the supply of growth substrates which are intermediates of the TCA cycle (i.e., citrate and succinate) or which are delivered to the TCA cycle, i.e., acetate, and odd-numbered organic acids (i.e., propionate or valerate), induced an increase of the fraction of odd-numbered fatty acids (both saturated and mono-unsaturated, with chain length of C15 and C17) in TAG compared to the growth on fructose or gluconate (Alvarez et al. 1997). Conversely, when the cells were cultivated on *n*-alkanes, the accumulated fatty acids were related to the carbon skeleton of the respective alkane used for growth. For instance, when *R. opacus* PD630 cells were cultivated on hexadecane as the sole carbon source, palmitic acid (C16:0) was the predominant fatty acid occurring in cells, whereas during PD630 growth on pentadecane, cells accumulated only odd-numbered fatty acids directly related to the carbon chain of the specific alkane and to its β-oxidation derivatives (Alvarez 2003). These results suggested that β-oxidation pathway does not only represent a catabolic route in oleaginous *Rhodococcus* spp. strains, but it is also a source of fatty acids. Further, the alkanes that are incorporated into cellular lipids are not completely degraded to acetyl-CoA (Alvarez 2003; Cappelletti et al. 2019b). These studies highlighted the possibility to drive lipid storage to specific branched-chain and odd-numbered fatty acids in *R. opacus* by only changing the growth substrate, making this species a valuable TAG producer for next-generation biofuels (Tsitko et al. 1999).

The limitation of the nitrogen source in the presence of an excess of carbon in the culture medium was reported to be the main trigger for TAG accumulation, causing an imbalance between C and N in cells, which redirects the carbon metabolism to lipogenesis (Alvarez et al. 2000). Early studies described the improved capacity of *R. opacus* PD630 to accumulate TAGs when the cells were growing under nitrogen limiting conditions (ca. 20 times less nitrogen than control) and in the presence of high amounts of gluconate (Alvarez et al. 2000). These results indicated that, in contrast to what was observed in many bacteria, PD630 cells do not block lipid metabolism under growth-limiting conditions and generate acyl-residues from the available carbon source leading to TAG production. Yet, TAGs were mobilized and used as carbon and energy source when *R. opacus* PD630 cells were incubated under carbon starvation conditions in the presence of a nitrogen source (Alvarez et al. 2000; Alvarez and Steinbüchel 2002; Alvarez 2019). Finally, TAG biosynthesis was dependent on the time of bacterial cultivation and, therefore, the growth phase. Indeed, *R. opacus* PD630 demonstrated shifts in the composition of TAGs during the progression from exponential to stationary phase under unbalanced growth conditions (Alvarez et al. 2000).

Due to the capability of *Rhodococcus* spp. strains to convert lignin-derived aromatic compounds (e.g., 4-hydroxybenzoate, benzoate, phenol, vanillate, guaiacol, and trans-*p*-coumaric acid, *p*-coumaric acids, cresol, and 2,6-dimethoxyphenol) into TAGs, members of this genus are promising microbial hosts for lignocellulosic biomass conversion into biofuels (Kosa and Ragauskas 2012; Bhatia et al. 2019). Diverse genetic, physiological, and biochemical studies along with more recent “omic” works provided indications on the metabolic pathways and regulatory mechanisms involved in the biosynthesis and accumulation of TAGs from different carbon sources, including lignocellulose biomasses (Chen et al. 2014; Dávila Costa et al. 2015). On the basis of these studies, genetic and metabolic engineering strategies were proposed to develop sustainable processes of biofuel production using *Rhodococcus* spp. strains, mostly *R. opacus* PD630 and *R. jostii* RHA1 (Castro et al. 2016; Anthony et al. 2019). In this context, *R. opacus* PD630 and *R. jostii* RHA1 cells expressing specific heterologous genes, e.g., *xylA* and *xylB* from *Streptomyces lividans* TK23, and *bglABC* operon from *Thermobifida fusca*, were shown to acquire the capacity to degrade cellulose, arabinose, and xylose from lignocellulosic biomass and to simultaneously produce lipids (Table 1) (Hetzler et al. 2013; Xiong et al. 2012, 2016a, 2016b; Hernández et al. 2015; Kurosawa et al. 2013). Through *A*daptive *L*aboratory *E*volution (hereafter: ALE) procedures, a series of *R. opacus* PD630 strains were generated, which were able to better utilize aromatics and produce lipids, to consume multiple carbon sources simultaneously (i.e., glycerol, glucose, and xylose in *R. opacus* MITGM-173, Table 1), and to better tolerate inhibitors (e.g., phenolic compounds), which are usually present in lignocellulosic hydrolysates (Kurosawa et al. 2015a, 2015b; Yoneda et al. 2016; Henson et al. 2018). Genome-based manipulation strategies based on CRISPR-Cas9 and recombineering, were successfully applied in *Rhodococcus* spp. strains, opening new frontiers for genetic and metabolic engineering of members of this genus aimed at optimizing biofuel production from lignocellulose bioconversion (DeLorenzo et al. 2018; Liang et al. 2020). In order to implement metabolic engineering strategies for waste bioconversion into valuable compounds, a genome-scale model was also developed in *R. jostii* RHA1 to describe and predict the accumulation rate of three types of carbon storage compounds (i.e., glycogen, polyhydroxyalkanoate, and triacyloglycerols) using different carbon sources (glucose or acetate) and under growth conditions typically occurring in activated sludge bioreactor systems for wastewater recovery (Tajparast and Frigon 2015, 2018).

### Polyhydroxyalkanoates

Polyhydroxyalkanoates (PHAs) are polyesters of various hydroxycarboxylic acids, which are produced by a variety of bacterial species generally under nutrient-restricting conditions in the presence of carbon in excess (Anderson and Dawes 1990). They are accumulated by bacteria as intracellular hydrophobic inclusions as carbon and energy storage or as electron sinks of redundant reducing power (Muhammadi et al. 2014). Accumulated PHAs are typically degraded by intracellular depolymerases and metabolized as carbon and energy sources as soon as the supply of the limiting nutrient is restored (Steinbüchel et al. 1992). Based on the type of monomer(s) constituent, PHAs can be homopolymers (e.g., polyhydroxybutyrate [PHB]), copolymers (e.g., poly(3-hydroxybutyrate-co-3-hydroxyvalerate [poly(3HB-co-3 HV) or PHBV]), or terpolymers (e.g., poly(3-hydroxybutyrate-co-3-hydroxyvalerate-co-3-hydroxyhexanoate) [P-3HB-co-3 HV-co-3HHx]) (Haywood et al. 1991). These polymers show good biodegradability, insolubility in water, non-toxicity, thermoplasticity, and/or elastomericity, which support their suitability for applications in the packaging industry, medicine, pharmacy, agriculture, food, and chemical industries (Anderson and Dawes 1990). Indeed, PHA is considered the green polymers of the future, since they are expected to gradually substitute conventional plastics (e.g., polypropylene (PP) and low-density polyethylene (LDPE)) with similar physicochemical, thermal, and mechanical properties (Kourmentza et al. 2017). Since the chemical synthesis of PHA is not feasible, microorganisms represent the only source for these polymers.

**Fig. 5:**
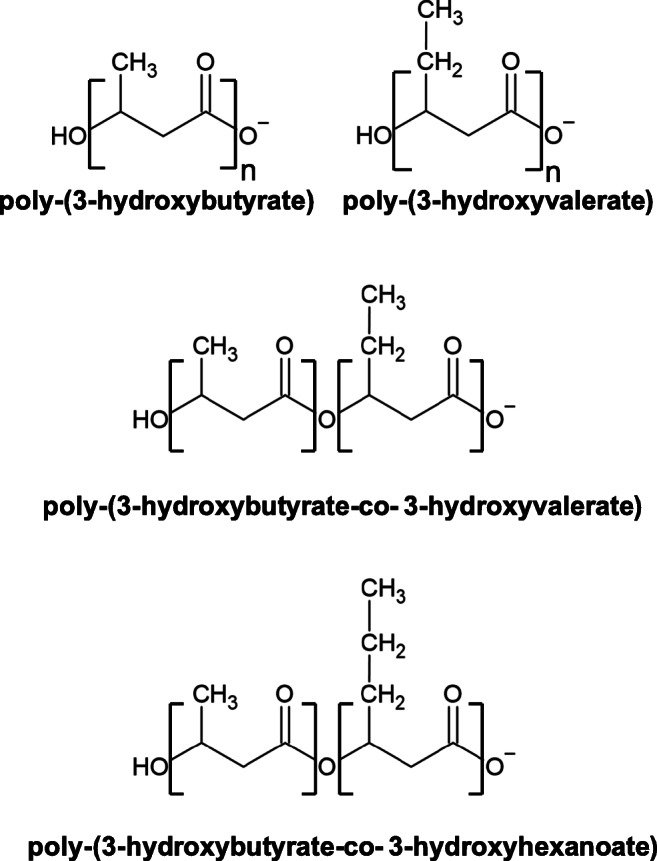
Chemical structures of the main polyhydroxyalkanoates (PHAs) produced by *Rhodococcus* spp. strains

Members of *Rhodococcus* genus are able to synthesize PHAs in variable amounts and composition depending on the bacterial strain and the carbon source (Haywood et al. 1991; Pieper and Steinbüchel 1992; Alvarez et al. 1997a,b; Alvarez and Steinbüchel 2002; Alvarez 2003) (Table 1, Fig. 5). One of the most extensively studied strains is *R. ruber* NCIMB 40126 which was found to be able to accumulate a peculiar PHA containing a copolymer of 3-hydroxyvalerate (3 HV) and 3-hydroxybutyrate (3HB) monomer units (i.e., poly(3HB-co-3 HV) or PHBV) upon cultivation under N-limiting conditions in the presence of different substrates such as acetate, lactate, succinate, fructose, glucose, and molasses (Table 1). In particular, this strain, as well as other members of genus *Rhodococcus* (e.g., *R. aetherivorans* IAR), showed the peculiar capacity to synthesize the commercially important PHBV from single carbon sources which were characterized by chemical structure unrelated with that of the copolymer (Hori et al. 2009)*.* The relative amount of each monomer changed depending on the carbon source, although HV was always predominant over HB (HV content range 60–91%) (Haywood et al. 1991). Several *Rhodococcus* strains, including NCIMB 40126 and *R. aetherivorans* IAR, NCIMB 40126 was also capable of incorporating 4HB and 5 HV monomer units into PHAs when provided with substrates which are precursors of these monomers (i.e., 4-hydroxybutyrate and 5-chlorovalerate, respectively) (Haywood et al. 1991). Further, this *R. ruber* strain accumulated almost pure poly(3-hydroxyvalerate) when supplied with valeric acid and 2-pentenoic acid, while when hexanoate or 2-hexenoate were used as substrates, a PHA containing 3HB, 3 HV, and 3-hydroxyhexanoate (3HHx) monomer units was produced (Haywood et al. 1991) (Fig. 5). In a different study performed by Alvarez et al. (1997b), *R. ruber* NCIMB 40126 showed higher accumulation of PHA than TAGs on valerate and glucose, whereas it seemed to accumulate only TAGs on hexadecane (Table 1) (Alvarez 1997b). *R. fascians* D 188–5, *R. erythropolis* DSMZ 43060, and *R. opacus* MR22 accumulated homopolymers of polyhydroxybutyrate (PHB) in addition to minor amounts of diacylglycerols and wax esters from various carbon sources, although the main carbon storage was TAG (Alvarez 1997b). *R. opacus* PD630 has been described to accumulate significant amounts of TAGs but not PHA from various carbon sources (Alvarez et al. 1996).

The possibility to combine biodegradation of toxic and contaminant molecules with PHA production was investigated to achieve a cost reduction of the biodegradable plastic production process, along with the effective utilization of wastes or toxic compounds. In this context, *R. aetherivorans* IAR was able to produce PHBV from toluene, which is the volatile organic compound (VOC) most abundantly emitted in the environment of some countries (e.g., Japan) (Hori et al. 2009). *R. aetherivorans* BCP1 accumulated electron-transparent intracellular inclusions composed by neutral lipids during the growth on naphthenic acid models (Presentato et al. 2018a). Although the chemical nature of these inclusion bodies is still undefined, recent experiments showed that, based on the different NAs used for growth, BCP1 cells accumulated either PHB or poly(3HB-co-3 HV) (Cappelletti, unpublished results).

Notably, IAR strain could also accumulate TAG along with PHA on toluene and acetate (Hori et al. 2009), being both PHBV and TAGs simultaneously synthesized and accumulated before the nitrogen source was exhausted. Subsequently, the accumulation of both the storage compounds continued, whereas only the TAG was synthesized even after the carbon source depletion, although at a low rate. This lipid production profile was different from that of *R. ruber* NCIMB 40126 cells growing on glucose, during which only the accumulation of PHBV started during the exponential growth phase (Alvarez et al. 2000). When the complete consumption of the nitrogen source occurred, PHBV content reached a maximum in NCIMB 40126 cells and the biosynthesis and accumulation of TAGs started. These results demonstrated that the accumulation of TAGs continued after carbon source exhaustion in both the strains, whereas, after substrate depletion, PHBV was mobilized and started to be degraded. With the aim of optimizing the bioprocess, this time course study gave indications on the culture and harvesting conditions that resulted in both high cellular content of these storage compounds and PHAs and TAGs of desired composition (Alvarez et al. 2000). The utilization of specific metabolic inhibitors led to the production of either PHA or TAG, defining possible selective biosynthesis strategies (Alvarez et al. 2000). These experiments also demonstrated that the biosynthetic route of PHA and fatty acids in *R. ruber* NCIMB 40126 compete for the common precursors, acetyl-CoA and propionyl-CoA, during cell growth under storage conditions (Alvarez et al. 2000).

PHA synthases represent the key enzymes of PHA biosynthesis, which catalyze the stereoselective conversion of (R)-3-hydroxyacyl-CoA substrates to PHA with the concomitant release of CoA (Hernández et al. 2008). The *phaC* gene from *R. ruber* is the only gene encoding for a PHA synthase identified and cloned from a member of *Rhodococcus* (Pieper and Steinbüchel 1992). This PhaC enzyme is a short-chain length class I PHA synthase, which comprises only one type of subunit that utilize CoA thioesters of 3-hydroxy fatty acids with 3 to 5 carbon atoms (Rehm 2003). Downstream of the *R. ruber phaC*, a gene coding for a putative PHA depolymerase (PhaZ) was identified, which is predicted to be the key enzyme for PHA mobilization. In the model strain *R. jostii* RHA1, three different chromosomal loci including the *phaC* and *phaZ* were recognized. Despite being similar to that described in *R. ruber*, this *pha* gene organization is different from that of other Gram-negative bacterial strains accumulating short-chain length PHA, which also typically include other genes involved in the PHA biosynthetic pathway such as *phaA* and *phaB* (Rehm and Steinbüchel 1999).

## Polyunsaturated fatty acids

Polyunsaturated fatty acids (PUFAs) are fatty acids containing more than two double bonds. Important PUFAs include omega-3 and omega-6 (ω-3 FA and ω-6 FA) fatty acids that are named based on the position of the first double bond from the methyl-end in the fatty acid chain. They are critical nutrients for human health, as they modulate brain development and cognition, as well as many diseases, such as cardiovascular disease, cancers, and diabetes (Lee et al. 2016). Furthermore, specific PUFAs are precursors of molecules regulating inflammatory and immune responses (Calder 2013). Humans must acquire PUFAs via foods or nutritional supplements because they do not contain delta-12 desaturases and convert molecules to PUFAs very slowly. Because of the nutraceutical and pharmacological importance, biotechnology methods have been used to clone and introduce numerous desaturases into diverse organisms to yield PUFAs with critical significance in diet. Many groups have investigated the use of marine algae as a commercial source (Patel et al. 2019); however, bioengineered bacterial strains may result in superior producers, due to the large biomass accumulation and high growth rate. In this context, *R. opacus* was proposed as a host strain of polyunsaturated fatty acid synthase genes (*pfa* genes from marine bacterial strain *Shewanella baltica*) expression for PUFA production (Blakie 2015), because of the ability of this species to accumulate high amount of fatty acids.

PUFA biosynthetic process has been mainly described in marine bacteria (Nichols 2003), but recently, *Rhodococcus erythropolis* and *R. aetherivorans* strains showed the ability to accumulate PUFAs, likely as a response mechanism associated with specific stress conditions, such as the exposition to a high salt concentration, extremely low temperatures, toxic metals, and the growth on toxic organic compounds (e.g., naphthenic acids) (de Carvalho 2012; de Carvalho et al. 2014; Presentato et al. 2018a). In particular, *R. aetherivorans* BCP1 cells accumulated significant amounts (around 7% of the total cell fatty acid content) of linoleic acid (omega-6, 9cis,12cis-C18:2) during the growth on the NA, cyclopentane carboxylic acid (CPCA). The molecular mechanisms supporting PUFA accumulation in *Rhodococcus* spp. strains have not been elucidated yet. Recent RT-qPCR experiments on RNA extracted from *R. aetherivorans* BCP1 cells grown on naphthenic acids did not clearly show the upregulation of any specific gene among those predicted to encode desaturases and polyketide synthases (Cappelletti, unpublished results).

## Antimicrobials

**Fig. 6:**
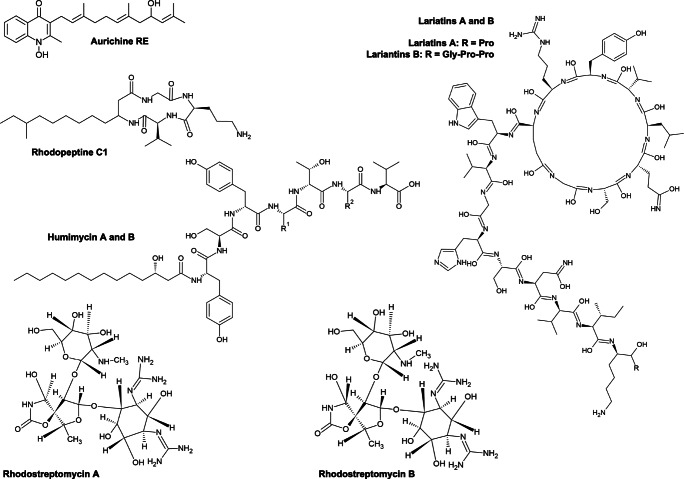
Chemical structures of the main antibiotics found to be produced up to date by *Rhodococcus* spp. strains

Antibiotics revolutionized the treatment and prevention of numerous types of infections and deadly diseases. As a side effect, many pathogenic bacteria have developed antibiotic resistance, which causes problems in the treatment and threatens modern healthcare. Increasing efforts are in progress to find new antibiotics or antimicrobial strategies to fight these strains. However, it is frequent to rediscover compounds that are already known, and this aspect presently slows down substantial developments in novel drug discovery. More than half of the presently known antibiotics have been isolated from *Streptomyces* spp. (Nepal and Wang 2019). Despite belonging to the same phylum (*Actinobacteria*), the knowledge on the production of antimicrobial compounds by *Rhodococcus* spp. strains is only a very recent matter of study (Kitigawa et al. 2018; Elsayed et al. 2017). Over the last 15 years, a few studies have reported this genus to be a good source of novel antibiotics (Table 1). To date, the lariantin peptide antibiotics, the polyketide aurachin RE, the rhodopeptins, and the humimycins are the natural and important products with antimicrobial activity that have been isolated from *Rhodococcus* strains (Fig. 6).

One of the first studies on antimicrobial production by *Rhodococcus* reported the strain *R. jostii* K01-B0171 to be able to produce lariantin A and B, which are antimycobacterial cyclic peptides consisting of 18 and 20 l-amino acid residues, respectively. They are defined as “lasso” peptides because of their structure having a macrolactam ring between the α-amino group of amino-terminal Gly1 and the γ-carboxyl group of Glu8 and a carboxy-terminal tail of the peptide looped back and threaded through the macrolactam ring (Fig. 1). These two peptides selectively inhibited the growth of *Mycobacterium smegmatis* and *Mycobacterium tuberculosis*, probably via targeting mycobacterial cell wall synthesis (Iwatsuki et al. 2006, 2007). The main biosynthetic gene cluster of the lariatins in *R. jostii* K01-B0171 was described to consist of five genes, *larABCDE*, in which *larA* encodes the precursor peptide which is post-translationally modified by LarB and LarD to produce lariatin. The *larE* gene encodes a possible ABC transporter involved in the mature lariatin export (Inokoshi et al. 2012).

**Fig. 7:**
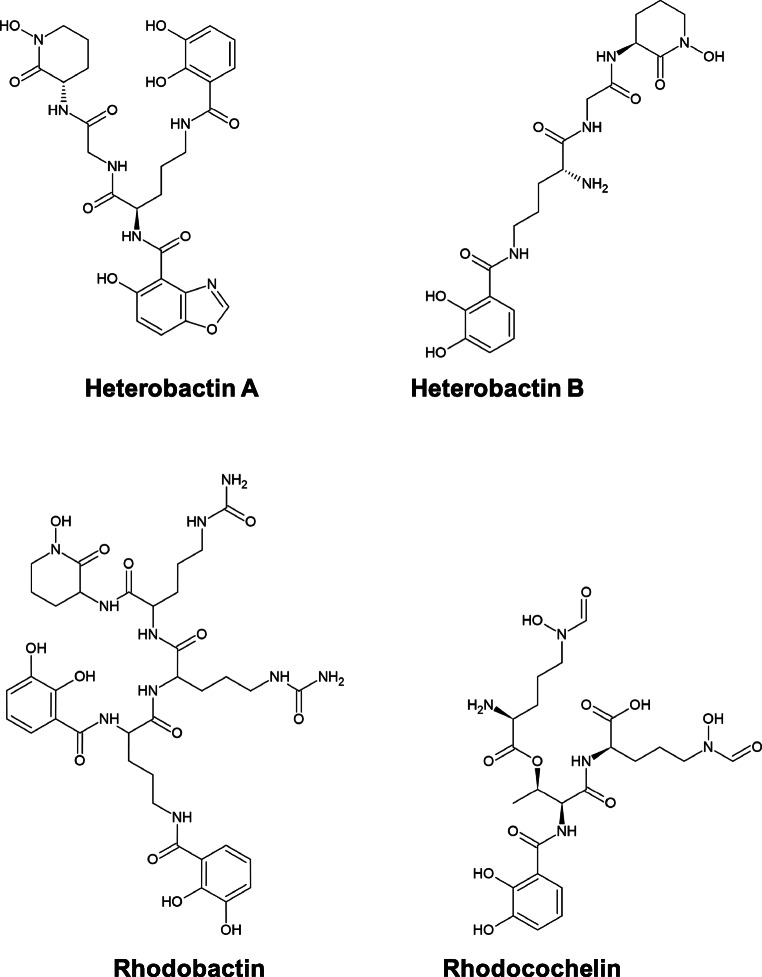
Structures of the main siderophores produced by *Rhodococcus* spp. strains (modified from Bosello et al. 2012)

By screening 80 *Rhodococcus* strains from several bacterial culture collections, Kitigawa et al. (2018) showed that 18 strains of five different *Rhodococcus* species had antimicrobial activities. Among these, a high number of strains belonging to *R. erythropolis* species were found to produce antibiotics. These *R. erythropolis* strains were divided into three groups depending on the antibiotic production (Kitigawa et al. 2018). In particular, one of the antibiotic-producing strains, *R. erythropolis* JCM 6824, was described to produce a new quinoline antibiotic aurachin RE which exhibited a strong activity against a broad range of Gram-positive bacteria. From a chemical point of view, aurachin RE has a structure very similar to aurachin C, which derives from the Gram-negative bacterium *Stigmatella aurantiaca* Sga15 (Fig. 7). However, compared to aurachin C, aurachin RE exhibited a wide and strong antimicrobial spectrum against both high- and low-GC Gram-positive bacteria (Kitagawa and Tamura 2008). Additional four aurachin molecules were isolated from *Rhodococcus* sp. Acta 2259 (Nachtigall et al. 2010). These compounds showed inhibitory activities against the growth of numerous Gram-positive bacteria, such as *Staphylococcus epidermidis* DSM 20044, *Bacillus subtilis* DSM 347, and *Propionibacterium acnes* DSM 1897, but not against Gram-negative bacteria (Nachtigall et al. 2010). Conversely, the cyclic tetrapeptide rhodopeptins that were first isolated from *Rhodococcus* sp. Mer-N1033 exhibited antifungal activity against *Candida albicans* and *Cryptococcus neoformis*, but they showed no antibacterial activity (Chiba et al. 1999).

The two antibiotics rhodostreptomycin A and B were instead isolated from culture broths of *R. fascians* 307CO cells after being subject to competitive co-culture experiments with the antibiotic producer *Streptomyces padanus*. These antibiotics were found to be biosynthesized following horizontal gene transfer of a large DNA segment derived from the *Streptomyces* strain. From a chemical point of view, rhodostreptomycins were identified as two isomers of a new class of aminoglycosides, which greatly differed in the structure from the *Streptomyces*-produced actinomycins (Kurosawa et al. 2008). Rhodostreptomycins exhibited good antibiotic activities against an extensive range of Gram-negative and Gram-positive bacteria, such as *Streptomyces padanus*, *Escherichia coli*, *Staphylococcus aureus*, *Bacillus subtilis*, and *Helicobacter pylori*. Rhodostreptomycin B was found to be more potent than rhodostreptomycin A, suggesting that their stereochemistry difference influenced the biological activity (Kurosawa et al. 2008) (Fig. 7).

Recently, the first bacteriocin-like molecule and its structural gene (*rap*) were obtained from *Rhodococcus erythropolis* JCM 2895. A 5.4-kb circular plasmid harbors *rapA* and *rapB* genes encoding for RapA and RapB proteins. RapA is a small, heat-stable, water-soluble protein showing antimicrobial activity against other *R. erythropolis* strains, while RapB is an immunity protein against RapA that may be located on the cell membrane (Kitigawa et al. 2018). Bacteriocins are usually peptides synthesized by ribosomes and have a narrow spectrum of activity; they are produced to inhibit the growth of competitor organisms in their environment to outcompete for nutrients (Gillor et al. 2008).

Differently to the other antibiotic compounds, the structure of humimycins was initially predicted through a bioinformatic approach (named syn-BNP, standing for synthetic-bioinformatic natural product), which scanned hundreds of bacterial genomes residing in the human body for clusters of genes that were likely to produce non-ribosomal peptides that form the basis of many antibiotics (Fig. 7) (Chu et al. 2016). Gene clusters encoding non-ribosomal peptide synthases producing humimycin A and B were detected from genomes of *Rhodococcus equi* and *R. erythropolis* strains. The genome of *Rhodococcus enclensis* NIO-1009 also showed a biosynthetic gene cluster coding for humimycin B (Ceniceros et al. 2016). The humimycin antibiotics are lipid II flippase inhibitors which hamper bacterial cell wall biosynthesis. They were found to be particularly effective against pathogenic and multidrug-resistant *Staphylococcus* and *Streptococcus* strains (Chu et al. 2016).

## Siderophores

Siderophores are low-molecular-weight compounds, which are released by some bacteria to chelate extracellular iron and transport it inside the cell. The production and release of siderophores allows bacteria to accumulate an intracellular pool of iron and to satisfy the nutritional iron requirement also under metal-limiting conditions (Kraemer 2004). Siderophores were also reported to bind other essential metals such as zinc, manganese, molybdenum, and vanadium for their acquisition, as well as to interact with heavy metals to prevent their cellular entry (Johnstone and Nolan 2015). Further siderophore functions include the protection against oxidative stress and the enhancement of antibiotic targeting and delivery (Johnstone and Nolan 2015). Based on the chemical structure, siderophores are classified as phenolate, hydroxamate, catecholate, (hydroxy-)carboxylate, and mixed types (Miethke and Marahiel 2007), and their biosynthesis occurs via different mechanisms (Miethke and Marahiel 2007). Areas of siderophore application include agriculture, medicine, pharmacology, bioremediation, biodegradation, and food industry. For instance, siderophores can be used to enhance plant growth due to their uptake by rhizobia, while in bioremediation strategies, siderophores can mobilize heavy metals and radionuclides (De Serrano 2017).

*Rhodococcus* spp. strains were described to be able to produce chemically diverse siderophores, such as rhodochelin, rhodobactin, heterobactin A, rhequichelin, and rhequibactin (Table 1 and Fig. 7). Carrano et al. (2001) were among the first groups to isolate a new class of siderophores, named heterobactins, from *R. erythropolis* IGTS8 culture. Among the three heterobactins that were produced by strain IGTS8, the two more abundant were named heterobactin A and B. From a structural point of view, heterobactins were catecholate-hydroxamate mixed-type siderophores, which contained both hydroxamate and catecholate donor groups. Heterobactins were also isolated from *R. erythropolis* PR4 by Bosello et al. (2013), who, through a bioinformatic analysis of the bacterial genome, identified the gene cluster responsible for heterobactin A’s biosynthesis. The biosynthesis of this siderophore was described to involve the activity of the modular enzymes nonribosomal peptide synthetases (NRPSs), in which each module adds a specific monomer to the peptide backbone. Similarly, a genomic study conducted on the other four *Rhodococcus* strains showed the presence of a high number of gene clusters encoding for NRPSs compared to other *Actinobacteria* (Doroghazi and Metcalf 2013).

Analogously to heterobactin, rhodochelin and rhodobactin belong to the hydroxamate–catecholate mixed type family and were isolated from *Rhodococcus jostii* RHA1 and *Rhodococcus rhodochrous* OFS, respectively (Dhungana et al. 2007; Bosello et al. 2011). While the gene clusters which determine rhodobactin synthesis were not identified, the gene clusters involved in the synthesis of rhodochelin were described in RHA1. This biosynthetic process involved the functional cross-talk between three distantly located NRPS gene clusters (Bosello et al. 2011), which were identified in most of *Rhodococcus* spp. strains (Bosello et al. 2011; Bosello et al. 2012).

Additional works assessed the capacity of siderophores produced by *R. erythropolis* S43 to bind trivalent arsenic, or arsenite [As(III)]. Interestingly, the arsenic-binding activity of the siderophore-like compounds from S43 was higher than the iron-chelating one. Although the capacity of the siderophore to bind and sequester toxic metals could not be clearly associated with arsenic resistance/tolerance mechanisms, the study provided the basis for a first analysis of different actinobacterial strains, including *R. erythropolis* S43, as a source of arsenic-binding compounds. When overproduced or in association with plants, this type of molecules could be used to decontaminate soil or water, as well as for other potential biotechnological applications (Retamal-Morales et al. 2018b).

## Metal(loid)-based nanostructures

Nanotechnology is defined as the world of “very small material” and it is based on the manipulation of matter at either molecular or atomic level (Horikoshi and Serpone 2013). The prefix *nano* is related to materials that feature at least one dimension in the nanorange (1–100 nm), where physical–chemical properties (i.e., high surface-to-volume ratio, large surface energy, and high spatial confinement) become enhanced as compared to their bulk counterparts (Cao 2004). Metal or metalloid (metal(loid)) nanostructures (NSs) have widespread applications in biomedicine/biotechnologies, energy production, environmental engineering, material science, and optoelectronics (Cao 2004; Horikoshi and Serpone 2013). Different physical–chemical methods efficiently produce high-quality nanomaterials (NMs) of various compositions and morphologies (including nanoparticles (NPs), nanocrystals (NCs), nanorods (NRs), nanowires (NWs), and nanotubes (NTs)) (Rao et al. 2004); however, these approaches mostly rely on dangerous operational conditions (e.g., high temperature and pressure), as well as the use of toxic and harsh chemicals (Zhang et al. 2006; CDC 2014). Moreover, there are still important challenges to be faced in this industry, among which the most urgent are represented by the generation of NSs featuring homogeneous size (i.e., monodispersity) and shape, as well as thermodynamic stability (Piacenza et al. 2018). The natural ability of microorganisms to cope with metal(loid) compounds, simultaneously generating NMs, has taken the form of an innovative and *eco-friendly* bioprocess.

**Fig. 8:**
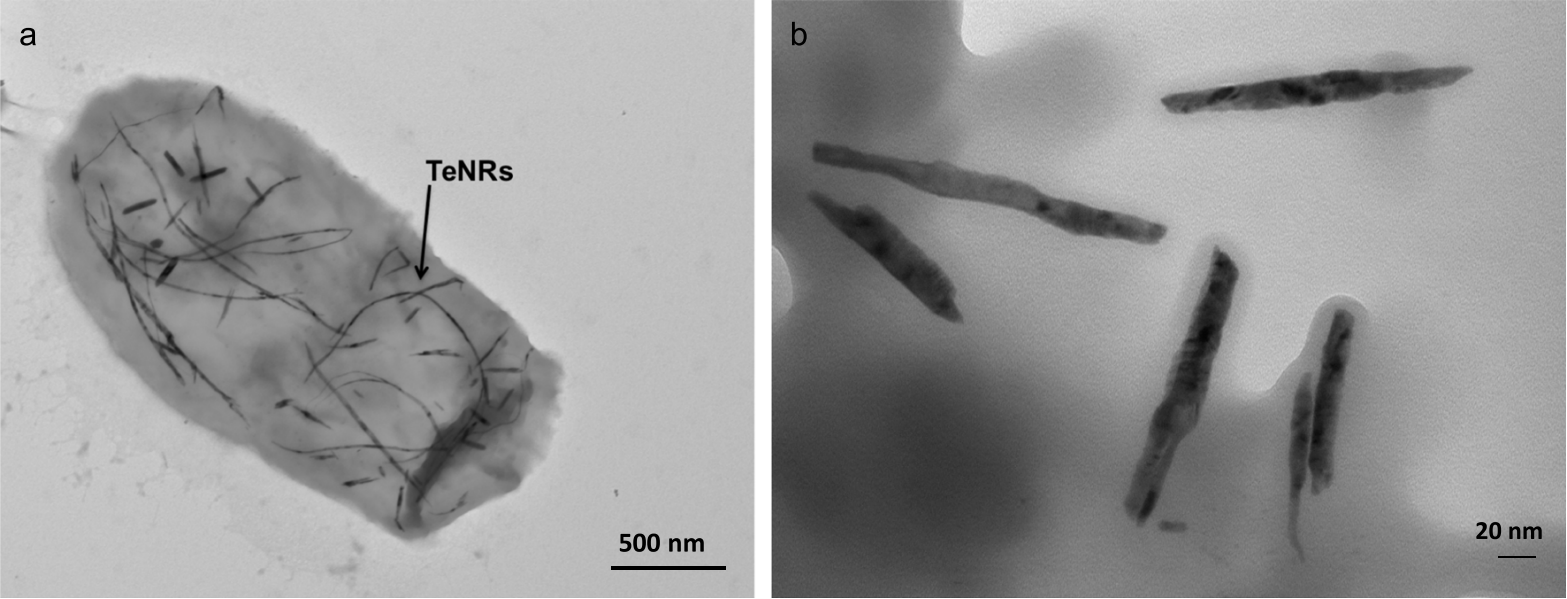
Tellurium-based nanorods (TeNRs) intracellularly produced by *Rhodococcus aetherivorans* BCP1 and visible inside the cell (**a**) or in the cell-free extract (after cell sonication) (**b**)

For the most part, bacterial cells cope and interact with large amounts of metal(loid) compounds by exploiting biosorption, bioaccumulation, and biotransformation processes, which are used for either detoxification or energy purposes (Li et al. 2011; Pantidos and Horsfall 2014). Bacteria belonging to the *Rhodococcus* genus tolerate the presence of various metal(loid)s, in many cases, due to their biotic conversion of metal(loid)s into a less toxic form. In this context, *Rhodococcus* spp. have been shown to bioconvert several metal(loid) precursors including gold (Au), silver (Ag), zinc oxide (ZnO), tellurium (Te), selenium (Se), and arsenic (As) synthesizing NMs, which are observed as either intra- or extracellular products, as a function of the strain and growth conditions (Fig. 8) (Ahmad et al. 2003; Otari et al. 2012; Subbaiya et al. 2014; Kundu et al. 2014; Presentato et al. 2016, 2018b, 2018c). The first study reporting rhodococci for metal(loid) NS production highlighted the ability of a *Rhodococcus* sp. strain to synthesize relatively monodisperse spherical AuNPs having ca. 12 nm as the average diameter and a good crystalline structure, as compared to chemogenic AuNMs (Ahmad et al. 2003). Similarly, *Rhodococcus* sp. NCIM2891 proved to be an efficient biocatalyst for the generation of intracellular, small (ca. 10 nm), spherical, and crystalline AgNPs (Otari et al. 2012), which were also described both as an antimicrobial, to be used in commercialized textiles and wound dressings, or an antitumor agent (Subbaiya et al. 2014). Iron oxide (Fe_2_O_3_) NPs were biosynthesized by *R. erythropolis* ATCC 427 cultures during biomining processes (Maas et al. 2019a, 2019b). In this context, parameters of the designed implants, as the stirring rate and oxygen flow rate, appeared to determine the size of the produced Fe_2_O_3_ NPs, allowing to obtain fairly monodisperse populations (50 or 100 nm), as well as α-Fe_2_O_3_ and β-Fe_2_O_3_ crystalline structures (Maas et al. 2019a, 2019b).

The role of rhodococci in the microbial nanotechnology field was deeper analyzed by Kundu and co-workers (2014), who focused on biosynthesis of extracellular ZnO NPs by *R. pyridinivorans* NT2. The produced ZnO NPs showed a *quasi*-spherical and hexagonal morphology, a large polydispersity (average size 100–120 nm), and a low tendency to aggregate. Nevertheless, these biogenic nanoproducts were as pure and crystalline as those commercially available, showing good absorbance and fluorescence properties (Kundu et al. 2014). Biogenic ZnONPs were also used as a coating for textile cotton surfaces, displaying good UV-blocking, self-cleaning, and antimicrobial properties (Kundu et al. 2014). Finally, these NPs resulted to be efficacious against colon carcinoma cells (HT-29), without showing cytotoxicity against normal peripheral blood mononuclear cells (Kundu et al. 2014).

**Fig. 9:**
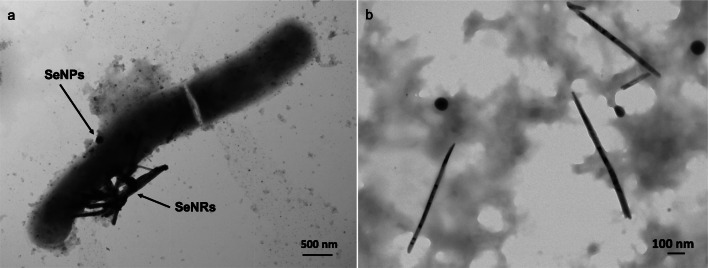
Selenium-based nanorods (NRs) and nanoparticles (NPs) produced by *Rhodococcus aetherivorans* BCP1 and visible on the cell membrane (**a**) or in the cell-free extract (**b**)

To date, *R. aetherivorans* BCP1 represents one of the most versatile and well-known strain belonging to this genus for NM biosynthesis. This fundamental technological trait relies, for the most part, on the ability of this bacterium to handle very high concentrations of the toxic oxyanions TeO_3_^2−^ and SeO_3_^2−^, biotically converting them in their elemental forms (Te^0^ and Se^0^) generating Te or Se NSs, such as NRs or NPs (Figs. 8 and 9). Moreover, by playing with the physiological state of BCP1 cells, as well as the amount of precursor concentration supplied to the culture medium, this strain could improve the average length of the intracellularly produced TeNRs, which featured a regular single-crystalline structure (Fig. 8) (Presentato et al. 2018c). These TeNRs also possessed an electrical conductivity comparable to chemogenic TeNMs, further supporting the technological value of Te-based nanoproducts synthesized by BCP1 cells (Presentato et al. 2018c). Curiously, exposing the same strain to SeO_3_^2−^, the production of SeNPs and NRs was associated with the cell membrane (Fig. 9) (Presentato et al. 2018b). Another distinctive case is represented by BCP1 incubated with increasing concentrations of arsenate [AsO_4_^3-^, also referred to as As(V)] in the presence of glucose as the sole carbon and energy source. Here, some preliminary TEM analyses showed the presence of electron-dense hexagonal nanoplates (possibly associated to metal(loid) biosorption) that deserve further analysis (Firrincieli et al. 2019).

The physical–chemical characterization of the metal(loid) NMs produced by *Rhodococcus* spp. also highlighted the absence of NS aggregation phenomena under diverse conditions, indicating their high thermodynamic stability and allowing to avoid post-production treatments of these biogenic NMs to ensure their high quality and applicability. This crucial feature seemed to rely on the presence of organic molecules derived from bacterial cells, which may simultaneously act as both electrostatic and steric stabilizers for the NMs in suspension (Piacenza et al. 2018). This aspect was deeply investigated for the ZnO NPs produced by *R. pyridinivorans* NT2 (Kundu et al. 2014), as well as biogenic Se or Te NMs synthesized by *R. aetherivorans* BCP1 (Presentato et al. 2016, 2018b, 2018c). Proteins and peptides were found to be important components of the biogenic NM extracts recovered from both rhodococci strains, as tyrosine (Kundu et al. 2014) or tryptophan residues (Presentato et al. 2016, 2018b, 2018c) were detected within the organic material recovered from NT2 or BCP1 cells, respectively. Additionally, intra and/or extracellular heterocyclic metabolites (in NT2 strain; Kundu et al. 2014) and intracellular amphiphilic biomolecules (in BCP1 strain; Presentato et al. 2016, 2018b, 2018c) remained associated with the NMs despite the cells were subjected to several washing steps.

Although rhodococci have been seen so far as good biocatalysts for the synthesis of metal(loid) NMs, yet the biochemical mechanism(s) behind their formation is still poorly understood. Some insights into these bioprocesses have been provided, including indications about biosorption phenomena deriving from electrostatic interactions between the charged metal(loid)s and enzyme functional groups located at the bacterial cell membrane level (Manimaran and Kannabiran 2017). Moreover, NAD(P)H-dependent reductases or secreted reductases were found to be responsible for the direct reduction of metal(loid) ions in different *Rhodococcus* strains (Manivasagan et al. 2016), including *Rhodococcus* sp. NCIM2891(Otari et al. 2012), *R. pyridinivorans* NT2 (Kundu et al. 2014), and *R. erythropolis* ATCC 427 (Maas et al., 2019). Painter-type reactions involving thiol-containing molecules (e.g., glutathiones and mycothiols) were suggested to mediate the intracellular or membrane-bound bioreduction of metalloid oxyanions, such as SeO_3_^2−^ and TeO_3_^2−^, by *R. aetherivorans* BCP1 (Presentato et al. 2016, 2018b, 2018c). In all cases, the bioreduction of metal(loid) precursor results in a highly localized concentration of elemental atoms, which would aggregate with each other to counteract their thermodynamic instability, and eventually assemble forming defined NSs (Ahmad et al. 2003; Adil et al. 2015). This phenomenon seems to be determined by both the thermodynamics of the metal(loid) element itself and the biomolecules in close proximity of the cluster forming elemental atoms (Piacenza et al. 2018). Indeed, the nature and the strength of the interaction occurring between proteins and/or specific enzymes with Au or Zn atoms appeared to control the size, shape, and crystallinity of the Au and ZnO NPs produced by *Rhodococcus* spp. (Ahmad et al. 2003) and *R. pyridinivorans* NT2 (Kundu et al. 2014). On the other hand, upon increasing the level of bioconversion of TeO_3_^2−^ within BCP1 cells, Te atoms collapse forming the so-called Te nucleation seeds, whose concentration is directly proportional to the oxyanion bioreduction extent. Once a critical concentration of Te seeds is reached in the intracellular environment, they aggregate firstly forming amorphous TeNPs, which, due to their relative thermodynamic instability, tend to dissolve and deposit along one preferential axis forming TeNRs, whose growth is likely mediated by the amphiphilic biomolecules present within the biogenic TeNM extracts (Presentato et al. 2018c).

## Conclusions and perspectives

Members of the *Rhodococcus* genus have been extensively described for their broad biodegradation activities and unique environmental persistence and robustness, mostly in association with their application in bioremediation strategies. During the last 20 years, the metabolic versatility of *Rhodococcus* spp. strains have been extended to their biosynthetic capabilities, which can find applications in industrial, environmental, and medical fields, including biofuel generation, metal biorecovery, and novel drug discovery. These biosynthetic pathways are often associated with cell response to challenging conditions of growth, e.g., starvation, hydrophobicity of the substrate and include (i) the production of biosurfactants or bioflocculants that facilitate the contact and metabolism of substrate compounds including organic contaminants, (ii) the generation of carotenoids to counteract oxidative stress, (iii) the accumulation of storage compounds (i.e., TAGs and PHAs), which is enhanced under nitrogen/carbon unbalanced growth conditions, (iv) the production of metal-based nanomaterials to detoxify toxic metal(loids), and (v) the biosynthesis of antimicrobials to outcompete other microorganisms. Some of the compounds produced by *Rhodococcus* (i.e., biosurfactants, bioflocculants, carotenoids) were demonstrated to have potential application to real industrial settings, due to their higher performance as compared to those produced by other organisms along with their lower toxicity as compared to the chemically synthesized counterpart. Additionally, the production of valuable compounds by *Rhodococcus* such as TAGs and PHAs could be associated with bioconversion of renewable feedstocks and low-cost wastes making it possible to exploit the metabolic versatility of members of this genus in sustainable and ecofriendly processes. In particular, much attention was paid to the study of lignocellulosic biomass conversion into TAGs by *R. opacus* PD630, which was proved to enhance to some extent through mutagenesis and genetic modification approaches.

Some challenges are well known and still ongoing for *Rhodococcus* strains used in industrial settings that are associated with the relatively slow growth, complex cell cycle, and low metabolic activity under non-optimal environmental conditions (Krivoruchko et al. 2019). Nevertheless, the application range of *Rhodococcus* biosynthetic processes has good chances to strongly increase in the near future thanks to the wide metabolic versatility and peculiar cell persistence under stress conditions of this bacterial genus. In this context, further research is required to expand our knowledge on regulatory mechanisms of *Rhodococcus* biosynthetic capacities in order to improve the production yields and efficiency but also to extend these studies to a larger number of members of this genus. Indeed *Rhodococcus* spp. are known to be extraordinarily diversified in terms of genomic contents, metabolic pathways, and evolutionary adaptations, even when considering strains of the same species (Cappelletti et al. 2019b). In this respect, different *Rhodococcus* spp. strains showed the ability to produce distinct compounds (also depending on the available substrates and the culture conditions) and comparative genomics studies highlighted the biosynthetic potentials of these bacteria that are still understudied (Ceniceros et al. 2017) which might predict new perspectives in terms of bioactive molecules identification and novel natural product discovery. Despite some knowledge is available on the major metabolic pathways involved in a few valuable compound bioproduction (e.g., TAG synthesis and accumulation in *R. opacus* PD630), our present knowledge on the genetic traits along with the metabolic pathways and regulation mechanisms of most of the biosynthetic activities in *Rhodococcus* spp. strains, is fragmentary (for biosurfactants and carotenoids), absent (for bioflocculants, antibiotics, metal-based nanostructures) or limited (for TAGs and PHAs) to only those strains, which are considered model strains of this genus (i.e., *R. opacus* PD630 and *R. jostii* RHA1) for their genetic tractability. In particular, more extensive multi-omic studies and metabolic engineering strategies of *Rhodococcus* strains are needed to delve into the major gene clusters involved in the anabolic pathways of interest and to identify specific genetic traits to be targeted for synthetic biology approaches. In this regard, novel genetic toolkits based on CRISPR/Cas9 system and recombineering have been recently applied for metabolic engineering of model strains of this genus (De Lorenzo et al. 2018; Liang et al. 2020), but they are mostly at the proof-of-concept stage and need to be further implemented to be used for the development of *Rhodococcus*-based microbial cell factories for efficient biotechnology applications. The extension of metabolic and genetic analyses of non-model *Rhodococcus* spp. strains and the advancements of synthetic biology tools for their genome-scale engineering will provide new opportunities for the use of an increasingly large number of strains of this genus in targeted bioconversion and biodegradation processes.
